# Evaluation of a *Bacillus*-based direct-fed microbial probiotic on in vitro rumen gas production and nutrient digestibility of different feedstuffs and total mixed rations

**DOI:** 10.1093/tas/txad044

**Published:** 2023-05-02

**Authors:** Bruno I Cappellozza, Jens N Joergensen, Giuseppe Copani, Keith A Bryan, Paolo Fantinati, Jean-Christophe Bodin, Mohammad Malek Khahi, Carlos NinoDeGuzman, Kathy G Arriola, Laís O Lima, Samia Farooq, Diwakar Vyas

**Affiliations:** Chr. Hansen A/S, Hørsholm 2970, Denmark; Chr. Hansen A/S, Hørsholm 2970, Denmark; Chr. Hansen A/S, Hørsholm 2970, Denmark; North America Technical Services, Chr. Hansen Inc., Milwaukee, WI 53214, USA; Chr. Hansen A/S, Hørsholm 2970, Denmark; Chr. Hansen A/S, Hørsholm 2970, Denmark; Department of Animal Sciences, University of Florida, Gainesville, FL 32611, USA; Department of Animal Sciences, University of Florida, Gainesville, FL 32611, USA; Department of Animal Sciences, University of Florida, Gainesville, FL 32611, USA; Department of Animal Sciences, University of Florida, Gainesville, FL 32611, USA; Department of Animal Sciences, University of Florida, Gainesville, FL 32611, USA; Department of Animal Sciences, University of Florida, Gainesville, FL 32611, USA

**Keywords:** *Bacillus* spp, digestibility, direct-fed microbials, forage, in vitro, neutral detergent fiber

## Abstract

We evaluated the effects of a *Bacillus*-based direct-fed microbial (**DFM**) on total in vitro gas production, dry matter (**DM**), neutral detergent fiber (**NDF**), and starch disappearance of different feedstuffs and total mixed rations (**TMR**) in three different experiments. In experiment 1, six single fiber-based feedstuffs were evaluated: alfalfa hay, buffalo grass, beet pulp, eragrostis hay, oat hay, and smutsvinger grass. Experimental treatments were control (with no probiotic inoculation; **CON**) or incubation of a probiotic mixture containing *Bacillus licheniformis* and *B. subtilis* (3.2 × 10^9^ CFU/g; DFM). The calculation of DFM dose under in vitro conditions was based on the assumption of a rumen capacity of 70 liter and the dose of 3 g of the DFM mixture/head/d (9.6 × 10^9^ CFU). Total in vitro gas production, DM, and NDF disappearance were evaluated at 24- and 48 h posttreatment incubation. Mean treatment effects were observed at 24- and 48 h gas production (*P* < 0.0001), as DFM incubation increased in vitro gas production by 5.0% and 6.5%, respectively. For nutrient digestibility, mean DM digestibility was increased at 48 h (*P* = 0.05), whereas mean NDF digestibility increased at both timepoints by incubating DFM in vitro (*P* ≤ 0.02). In experiment 2, nine commercial dairy TMR were collected and evaluated for the same variables and treatments described in experiment 1, with the additional analysis of starch digestibility at 7 h post in vitro incubation. The only difference was the concentration of the DFM included, being representative for a dosage of 8.8 × 10^9^ CFU/head/d. In vitro gas production was increased only at 48 h due to DFM incubation (*P* = 0.05), whereas DM and NDF digestibility were improved at 24 and 48 h (*P* ≤ 0.02). No treatment effects were observed on in vitro starch digestibility (*P* = 0.31). In experiment 3, a combined analysis of DM and NDF digestibility was performed by using quality values (NDF and crude protein or CP) of 16 substrates. Regardless of CP and NDF levels of the substrates, DFM improved in vitro 24 and 48 h DM and NDF digestibility (*P* ≤ 0.03). In summary, incubating a *Bacillus*-based DFM (*B. licheniformis* and *B. subtilis;* BOVACILLUS) improved mean in vitro gas production, DM, and NDF digestibility of single feedstuffs and commercial dairy TMR, highlighting the potential of this combination of *Bacillus* spp. to improve nutrient utilization, mainly fiber.

## INTRODUCTION

The use of direct-fed microbials (**DFM**) has been gaining attention due to their benefits on the health and performance of the dairy and beef cattle herd (FAO/WHO, 2001; Krehbiel et al., 2003; McAllister et al., 2011), besides also potentially replacing antibiotics and reducing public scrutiny against livestock production. More specifically, several types of DFM, or probiotics, have been evaluated under different production settings for young and mature ruminants, but increasing interest has been given to *Bacillus* spp., as its different modes of action warrant potential benefits as probiotics for humans and livestock species (Dias et al., 2022; Luise et al., 2022).

Bacilli are gram-positive, spore-forming, aerobic, and facultative anerobic bacteria (Bernardeau et al., 2017) and until now, more than 2,700 species of *Bacillus* spp. have been recognized (www.lpsn.dsmz.de). Recently, Pan et al. (2022) reported that a mixture of *B. licheniformis* and *B. subtilis* improved in vitro dry matter (**DM**), neutral detergent fiber (**NDF**), and starch digestibility of forage-based and concentrate feedstuffs originated from Australia, indicating that the ability of *Bacillus* spp. to produce and release a wide type and quantity of enzymes might promote feedstuff or nutrient utilization (Schallmey et al., 2004; Elshaghabee et al., 2017; Luise et al., 2022) in ruminants. However, additional research efforts are warranted to understand its effects on in vitro nutrient digestibility of forage-based feedstuffs from other regions often used in ruminant diets and from commercial dairy total mixed rations (**TMR**). Hence, we hypothesized that incubation of a combination of *Bacillus* spp. would improve in vitro nutrient digestibility in forage-based feedstuffs and from commercial dairy TMR obtained from North America. Therefore, our objective was to evaluate the effects of *Bacillus* spp. incubation on in vitro gas production and nutrient digestibility of individual feedstuffs (experiment 1) and commercial dairy TMR (experiment 2), as well as to perform a combined analysis with feedstuffs previously used by Pan et al. (2022; experiment 3).

## MATERIALS AND METHODS

### Experiment 1: Single Feedstuffs

This experiment was conducted at the University of Florida (Gainesville, FL) from May to November 2022.

Six individual feedstuffs originated from South Africa and China were evaluated in the present study: alfalfa hay (*Medicago sativa*), buffalo grass (*Bouteloua dactyloides*), beet pulp, eragrostis hay (*Eragrostis curvula*), oat hay (*Avena sativa*), and smutsvinger grass (*Digitaria eriantha*). All feedstuffs were analyzed in duplicates by wet chemistry procedures for concentrations of crude protein [**CP**; method 984.13; AOAC (2006)], soluble CP (Licitra et al., 1996), neutral detergent fiber [**NDF**; Van Soest et al. (1991); modified for use in an Ankom-200 fiber analyzer; Ankom Technology Corp., Fairport, NY], acid detergent fiber [**ADF**; method 973.18 modified for use in an Ankom-200 fiber analyzer; Ankom Technology Corp.; AOAC (2006)], and lignin (Van Soest, 1973). Moreover, total digestible nutrient (**TDN**) concentration was calculated according to equations proposed by NRC (2001). The nutritional profile of the substrates evaluated herein is presented in Table 1.

**Table 1.** Nutritional profile of the feedstuffs used in experiments 1 and 2

Item^1^	DM, % as-fed	CP, % DM	Soluble CP, % CP	NDF, % DM	ADF, % DM	Lignin, % DM	Starch, % DM	TDN, % DM
Experiment 1								
Alfalfa hay	90.8	24.4	39.5	32.1	24.7	5.2	–	69.4
Beet pulp	91.3	6.4	–	41.6	–	–	–	87.7
Buffalo grass	91.2	13.3	36.6	64.6	38.5	3.8	–	58.5
Eragrostis hay	90.7	9.1	29.0	74.2	42.6	6.0	–	55.2
Oat hay	91.4	5.6	35.1	60.1	37.8	5.9	–	59.0
Smutsvinger grass	90.1	9.8	27.3	66.2	45.5	7.9	–	53.0
Experiment 2								
TMR #1	92.5	18.1	41.3	26.1	18.8	2.2	27.0	75.1
TMR #2	92.2	17.1	45.0	29.4	22.5	3.1	25.4	72.7
TMR #3	92.6	17.8	37.9	34.1	24.8	3.0	19.8	70.0
TMR #4	92.8	17.6	47.1	32.2	24.8	3.9	25.0	69.6
TMR #5	92.9	18.1	41.6	30.0	22.1	3.2	20.4	71.7
TMR #6	93.5	18.9	36.8	25.1	19.6	3.4	26.6	73.8
TMR #7	93.1	17.5	42.9	27.9	20.2	2.8	21.0	73.6
TMR #8	93.0	17.6	42.0	28.0	20.3	9.3	19.1	72.8
TMR #9	92.9	17.6	38.8	25.6	20.0	12.6	26.4	73.4

^1^ **DM**, dry matter; **CP**, crude protein; **NDF**, neutral detergent fiber; **ADF**, acid detergent fiber; **TDN**, total digestible nutrients.

The experimental treatments evaluated herein were a control (no probiotic inoculation; **CON**) or inoculation of a mixture of a DFM containing *Bacillus licheniformis* and *B. subtilis* (3.2 × 10^9^ CFU of the mixture/g; BOVACILLUS, Chr. Hansen A/S, Hørsholm, Denmark; **DFM**) into the jars containing the rumen fluid of lactating dairy cows. The calculation of the DFM dose to be incubated into each jar was based on a rumen capacity of 70 liter and the dose of 3 g of the DFM mixture/head/d (total dose of 9.6 × 10^9^ CFU/head/d). Additionally, three independent runs were performed herein.

#### Donor animals and inoculum collection.

 Three-rumen fistulated lactating Holstein cows were used as the inoculum source for the present study. The donor cows were fed ad libitum twice daily (0600 and 1400 hours) a total mixed ration (**TMR**) that contained (dry matter basis) 38.2% corn silage, 27.3% ground corn, 14.5% soybean meal, 9.2% citrus pulp, 5.0% mineral mixture and clay, and 1.8% calcium salts of fatty acids. Between 2 to 3 h postfeeding, rumen fluid was manually collected, filtered through a two-layer cheesecloth, and pooled into prewarmed thermos flasks kept at 39 °C. The dairy cow TMR did not contain any other feed additive such as prebiotics, probiotics, enzymes, ionophores, and/or nonionophores (virginiamycin, for example). Thermo flasks containing pooled rumen fluid were kept airtight until transported to the laboratory for final filtration into two more layers of cheesecloth (total of four layers).

#### In vitro batch culture experiment.

Individual feedstuffs (1 mm, 0.5 g) were dried in the lab at 105 °C for 24 h and then weighed into Ankom F-57 filter bags (Ankom Technology, Macedon, NY). Before sample weighing, empty F-57 filter bags were prerinsed in acetone for 5 min and then air dried per manufacturer instructions. Bags were sealed with an Uline Tabletop Poly Bag Sealer (Impulse type AIE-200, American International Electronics, Kenneth City, FL) and immediately placed into 160 mL serum vials.

The inoculum collected from each cow was pooled and added to a buffered prewarmed (39 °C) media (Goering and Van Soest, 1970) in a 1:2 ratio of rumen fluid:artificial saliva (McDougall, 1948). The media was continuously infused with carbono dioxide (**CO**_**2**_) to maintain an anaerobic environment for the rumen fluid inoculum. Buffered rumen fluid (52 mL) was added to the 160 mL serum vials containing Ankom bags and a continuous stream of CO_2_ was flushed into the vials during the whole inoculation process. Vials were closed with rubber stoppers and crimped with aluminum seals. Vials were immediately placed in an air-forced incubator at 39 °C with a shaking system for 24 h for the study.

#### In vitro dry matter (IVDMD) and neutral detergent fiber (IVNDFD) digestibility.

The blank correction was used to estimate net gas production as well as for IVDMD and IVNDFD. Incubations were terminated by placing the bottles on ice after 24 h of incubation, with bags being taken out of vials, washed with tap water, and then dried in a forced-air oven at 60 °C for 48 h. Dried residues were weighed and the remaining amount was used to estimate apparent IVDMD. Bags were then analyzed for aNDF using sodium sulfite and heat-stable α-amylase in an Ankom-200 fiber analyzer (Ankom Technology Corp.). Then, bags were dried once again at 60 °C for 48 h and weighed to calculate IVNDFD.

#### In vitro gas production.

 Serum flasks were independently incubated for 24 and 48 h of incubation and headspace gas pressure was measured after the incubation period (at 24 and 48 h only). Gas pressure was corrected for gas pressure in blank flasks and converted to gas volume based on the current lab conditions, using the following equation:


$$\begin{array}{l} GV\left(mL\right)=\left(GP\;\times \;4.8843\right)+3.1296;\;\;r^{2}=0.97, \end{array}$$

where, GV = gas volume (in mL) and GP = gas pressure (in psi).

### Experiment 2: Commercial Dairy Total Mixed Rations (TMR)

This study was conducted at the University of Florida (Gainesville, FL) from June 2022 to January 2023.

Nine commercial dairy TMR were collected from seven different states of U.S., including California, Iowa, New Mexico, North Carolina, Texas, Virginia, and Wisconsin. The nutritional profile of the commercial TMR is presented in Table 1. The experimental treatments evaluated herein were the same as reported in experiment 1 (CON and DFM), following the same rationale for calculation of the dose to be incubated (4 g/head of a mixture containing *B. licheniformis* and *B. subtilis*; 2.2 × 10^9^ CFU of the mixture/g; BOVACILLUS, Chr. Hansen Inc., Milwaukee, WI) into the jars containing the rumen fluid of the animals. Additionally, three independent runs were performed herein.

#### Donor animals and inoculum collection.

The procedure for collection and nutritional management of the donor lactating Holstein cows was similar to what has been presented and described in experiment 1. The TDN concentration of the TMRs was calculated according to NRC (2001).

#### In vitro dry matter (IVDMD) and neutral detergent fiber (IVNDFD) digestibility.

The same procedure and number of replicates used in experiment 1 were applied to experiment 2, evaluating both IVDMD and IVNDFD at 24 and 48 h.

#### In vitro starch digestibility (IVSD).

A 7 h single incubation time with substrate ground at 4-mm was chosen for this study as it has been adopted by most of the commercial laboratories. Recently, Ferreira et al. (2018) estimated that mean ruminal starch half-life of corn grain was 6.6 h. Therefore, based on this finding, the present authors believe that using the 7 h timepoint would give us an accurate and adequate indication of in vitro starch degradability. After 7 h of incubation, bags were removed and placed immediately on ice to stop the fermentation, rinsed in a washing machine, dried at 60 °C for 48 h, and residues were used for starch analysis. Starch analysis was conducted on samples after pre-extracting for sugar by incubating in a 40 °C water bath followed by filtration on Whatman filter paper no. 41 (Cytiva Life Sciences, Malborough, MA). Residues were solubilized using an autoclave followed by incubation with glucoamylase enzyme to hydrolyze starch to produce dextrose (glucose). Prepared samples were injected into sample chamber of YSI Analyzer (YSI-2700 SELECT Biochemistry Analyzer, Yellow Springs, OH), where dextrose diffuses into a membrane containing glucose oxidase. The dextrose was immediately oxidized to hydrogen peroxide (**H**_**2**_**O**_**2**_) and D-glucono-4-lactone, with H_2_O_2_ being detected amperometrically at the platinum electrode surface. The current flow at the electrode was directly proportional to the hydrogen peroxide concentration, and hence to the dextrose concentration. Starch was determined by multiplying dextrose by 0.9 (Method adapted from Dairy One laboratory, Ithaca, NY).

### Experiment 3

Following experiment 1., data from this and our previous in vitro research study (Pan et al., 2022) were compiled into a larger dataset (*N* = 16 forage-based feedstuffs) to understand how *Bacillus*-based DFM would impact in vitro nutrient digestibility (IVDMD and IVNDFD). Of note, Pan et al. (2022) evaluated 10 different single forage-based feedstuffs inoculated or not with the same *Bacillus*-based DFM (BOVACILLUS; Chr. Hansen A/S) evaluated herein. Briefly, the feedstuffs were classified as high- and low-quality based on the median value of NDF (high-quality: ≤ 61.6%; low-quality: ≥ 64.2%) of the 16 feedstuffs included in this analysis. The same rationale of nutritional and quality classification was applied to the CP content of the feedstuffs (high-quality: ≥ 9.8%; low-quality: ≤ 9.5%).

#### Statistical Analysis.

All analysis were performed using the SAS software (version 9.4; SAS Inst. Inc.; Cary, NC). For experiments 1 and 2, the PROC MIXED of SAS was used and the Satterthwaite approximation to determine the denominator df for the test of fixed effects. Statistical model for gas production, IVDMD, IVNDFD (experiments 1 and 2), and IVSD (experiment 2 only) included the effects of treatment only. Furthermore, individual feedstuffs evaluated in experiment 3 were initially analyzed with the PROC MIXED procedure of SAS (v.9.4; SAS Inc.), using treatment, quality [low (**L**) or high (**H**) based on CP and NDF content of the feedstuffs], and interaction as fixed effects. Additionally, a Pearson correlation analysis was performed in experiment 3 using the PROC CORR of SAS (v. 9.4; SAS Inc.). Significance was declared at *P* ≤ 0.05 and tendencies were denoted if *P* > 0.05 and *P* ≤ 0.10.

## RESULTS

### Experiment 1

Mean in vitro gas production at 24 h was 5.0% greater, while mean IVNDFD was 12.8 % greater (*P* < 0.01; Table 2) ­following DFM inoculation with substrates in the rumen fluid. However, no treatment effects were observed on 24 h mean IVDMD (*P* = 0.26). At 48 h, a 6.5% improvement was observed in mean in vitro gas production when DFM was inoculated in experiment 1 (*P* < 0.01; Table 2). Conversely to DM digestibility at 24 h, DFM inoculation improved 48 h in vitro DM and NDF digestibility vs. CON (*P* ≤ 0.05; Table 2), with improvements of 3.7% and 15.2%, respectively.

**Table 2.** Mean in vitro gas production, dry matter (**DM**), and neutral detergent fiber (**NDF**) digestibility at 24 and 48 h of single feedstuffs (*N* = 6) incubated or not (**CON**) with a *Bacillus*-based direct-fed microbial (**DFM**) in experiment 1^1^

Item	Treatments		SEM	*P*-values
	CON	DFM		
24 h				
Gas production, mL/g dry matter	72.8	76.5	0.53	< 0.01
Dry matter digestibility, %	45.2	45.9	0.41	0.26
Neutral detergent fiber digestibility, %	24.6	27.7	0.87	0.02
48 h				
Gas production, mL/g dry matter	95.1	101.3	1.41	< 0.01
Dry matter digestibility, %	57.0	59.1	0.75	0.05
Neutral detergent fiber digestibility, %	39.2	45.1	1.29	< 0.01

^1^ **DFM**, *Bacillus*-based direct-fed microbial incubated in the rumen fluid (*B. licheniformis* and *B. subtilis*; BOVACILLUS, Chr. Hansen A/S).

Gas production was increased for alfalfa hay, beet pulp, eragrostis hay, and smutsvinger grass after 24 h inoculation with DFM (*P* ≤ 0.01), whereas similar results tended to be observed for 24 h DM digestibility of alfalfa hay and beet pulp (*P* = 0.09; Table 3). At 48 h, mean in vitro gas production was greater following DFM inoculation in alfalfa hay and beet pulp (*P* ≤ 0.04; Table 3). Similarly, 48 h in vitro DM digestibility was greater in beet pulp inoculated with DFM (*P* = 0.03), whereas NDF digestibility was greater in beet pulp (*P* ≤ 0.02) and tended to be greater for alfalfa hay (*P* ≤ 0.10; Table 3).

**Table 3.** In vitro gas production (mL/g dry matter), dry matter (**DM**), and neutral detergent fiber (**NDF**) digestibility (%) at 24 and 48 h of single feedstuffs (*N* = 6) incubated or not (**CON**) with a *Bacillus*-based direct-fed microbial (**DFM**) in experiment 1^1^^,^^2^

Item	Gas production			In vitro DM			In vitro NDF		
	CON	DFM	SEM	CON	DFM	SEM	CON	DFM	SEM
24 h									
Alfalfa hay	70.7^b^	78.4^a^	0.91	56.6^b^	58.3^a^	0.85	24.9	28.4	0.67
Beet pulp	105.0^b^	107.2^a^	1.22	57.4	58.2	0.63	23.5	26.0	0.73
Buffalo grass	73.0	75.1	1.59	41.8	43.7	1.77	30.9	33.2	1.85
Eragrostis hay	54.0^b^	58.8^a^	0.95	27.6	30.0	0.71	19.7	22.2	0.85
Oat hay	84.6	83.6	1.48	52.1	49.3	0.56	24.9	29.0	3.64
Smutsvinger grass	49.1^b^	55.8^a^	1.33	35.2	35.7	0.86	23.4	27.6	2.51
48 h									
Alfalfa hay	98.3^b^	108.7^a^	3.47	69.4	73.0	2.04	37.5	46.8	2.78
Beet pulp	137.2^b^	151.6^a^	2.93	71.7^b^	78.3^a^	2.34	45.6^b^	64.0^a^	3.36
Buffalo grass	90.6	95.7	4.95	57.1	58.6	2.55	47.8	49.7	3.09
Eragrostis hay	70.7	72.1	1.89	40.6	38.1	1.06	30.1	28.2	0.97
Oat hay	106.6	109.4	4.99	59.7	62.1	1.64	44.6	52.0	5.92
Smutsvinger grass	67.3	70.4	1.30	43.4	44.6	0.44	29.2	30.4	0.53

^1^ **DFM**, *Bacillus*-based direct-fed microbial incubated in the rumen fluid (*B. licheniformis* and *B. subtilis*; BOVACILLUS, Chr. Hansen A/S).

^2^Different letters on the same row denote differences at *P* < 0.05 level.

### Experiment 2

No treatment effects were observed on mean 24 h in vitro gas production (*P* = 0.53; Table 4), but an 9.2% improvement on 24 h gas production was observed in TMR #6 (*P* = 0.02; data not shown) inoculated with DFM. Overall, 24 h DM and NDF digestibility were greater for DFM-inoculated TMR vs. CON diets (*P* ≤ 0.02; Table 2). Moreover, TMR#8 and #9 had a greater DM digestibility, whereas NDF digestibility was greater for TMR#2 and #5 (*P* ≤ 0.04) and tended to be greater for TMR#4 (*P* = 0.07) inoculated with DFM (data not shown).

**Table 4.** Mean in vitro gas production, dry matter (**DM**), and neutral detergent fiber (**NDF**) digestibility at 24 and 48 h of commercial dairy total mixed rations (**TMR**; *N* = 9) incubated or not (**CON**) with a *Bacillus*-based direct-fed microbial (**DFM**) in experiment 2^1^

Item	Treatments		SEM	*P*- values
	CON	DFM		
24 h				
Gas production, mL/g dry matter	79.1	79.7	0.72	0.53
Dry matter digestibility, %	64.9	66.1	0.29	0.01
Neutral detergent fiber digestibility, %	21.2	23.7	0.48	< 0.01
48 h				
Gas production, mL/g dry matter	104.7	107.7	1.06	0.05
Dry matter digestibility, %	65.7	67.5	0.55	0.03
Neutral detergent fiber digestibility, %	33.2	37.2	0.63	<0.001

^1^ **DFM**, *Bacillus*-based direct-fed microbial incubated in the rumen fluid (*B. licheniformis* and *B. subtilis*; BOVACILLUS^TM^, Chr. Hansen Inc.).

Mean 48 h in vitro gas production was greater in TMR with DFM vs. CON (*P* = 0.05; Table 4). When DM and NDF digestibility was analyzed at 48 h, mean in vitro digestibility of both nutrients were greater following DFM treatment (*P* ≤ 0.03; Table 4) and improvements on in vitro NDF digestibility were also observed or tended to be observed for TMR#2, #5, and #6 (*P* ≤ 0.06; data not shown).

Lastly, no treatment effects were observed on IVSD (*P* = 0.31; 62.1% vs. 62.9% for CON and DFM, respectively; SEM = 0.49). However, TMR#6 had a greater 7 h IVSD following treatment with DFM vs. CON (*P* = 0.02; 56.2% vs. 62.0% for CON and DFM, respectively; SEM = 1.74).

### Experiment 3

Following the classification based on median CP and NDF content of the feedstuffs used in experiment 1 and in Pan et al. (2022), no CP and NDF class interactions were observed (*P* > 0.11). Regardless of CP and NDF classification (quality), DFM improved in vitro 24- and 48 h DM and NDF digestibility (*P* ≤ 0.03), whereas NDF content also impacted 24- and 48 h DM digestibility (*P* < 0.01; Table 5).

**Table 5.** Mean in vitro dry matter (**DM**) and neutral detergent fiber (**NDF**) digestibility of the feedstuffs (*N* = 16) incubated or not (**CON**) with a *Bacillus*-based direct-fed microbial (**DFM**) that were used herein and by Pan et al. (2022)^1^

Item	Treatments		SEM	*P-*value
	CON	DFM		
In vitro DM digestibility, %				
24 h	38.0	41.5	2.69	0.03
48 h	57.0	61.3	2.71	0.01
In vitro NDF digestibility, %				
24 h	18.9	24.4	2.41	0.01
48 h	42.5	49.5	3.33	< 0.01

^1^ **DFM**, *Bacillus*-based direct-fed microbial incubated in the rumen fluid (*B. licheniformis* and *B. subtilis*; BOVACILLUS, Chr. Hansen A/S).

For the CON samples, negative correlations were observed between NDF content of the feedstuff and in vitro DM digestibility at 24 and 48 h (*P* ≤ 0.02; Table 6), whereas positive correlations were observed between 24 and 48 h DM, 24 h DM and NDF, 48 h DM and 24 h, and 48 h DM and NDF digestibility (*P* ≤ 0.04; Table 6). Similarly, negative correlations were observed between NDF content of the feedstuff and in vitro DM digestibility at 24 and 48 h (*P* ≤ 0.02; Table 6) in DFM-inoculated samples. Moreover, 24 h DM digestibility positively correlated with 24 h NDF, as well as 48 h DM and NDF digestibility (*P* < 0.01), 48 h DM digestibility correlated with 48 h NDF digestibility, and 24 h NDF digestibility also correlated with 48 h NDF digestibility (*P* ≤ 0.04, Table 6).

**Table 6.** Pearson correlation between feedstuff crude protein (**CP**), neutral detergent fiber (**NDF**), in vitro dry matter (**DM**), and NDF digestibility in feedstuffs (*N* = 16) incubated or not (**CON**) with a *Bacillus*-based direct-fed microbial (**DFM**) in experiment 3

	CP, %	NDF, %	24 h DMD, %	48 h DMD, %	24 h NDFD, %
CON					
NDF, %	−0.672				
	<0.01				
24 h DMD, %	0.305	−0.736			
	0.25	<0.01			
48 h DMD, %	0.179	−0.594	0.763		
	0.51	0.02	<0.001		
24 h NDFD, %	0.159	−0.320	0.791	0.512	
	0.56	0.23	<0.001	0.04	
48 h NDFD, %	−0.199	0.004	0.372	0.783	0.379
	0.46	0.99	0.16	<0.001	0.15
DFM					
NDF, %	−0.672				
	<0.01				
24 h DMD, %	0.243	−0.666			
	0.36	<0.01			
48 h DMD, %	0.464	−0.769	0.831		
	0.07	<0.001	<0.0001		
24 h NDFD, %	−0.087	−0.038	0.728	0.427	
	0.75	0.89	<0.01	0.10	
48 h NDFD, %	0.186	−0.392	0.664	0.874	0.514
	0.49	0.13	<0.01	<0.0001	0.04

## DISCUSSION

The main goal of the present experiment was to evaluate the effects of a *Bacillus*-based direct-fed microbial on in vitro gas production, DM, NDF, and starch digestibility of different individual feedstuffs and commercial dairy TMR. Previous literature reviews and research studies have demonstrated the importance of optimizing rumen nutrient digestibility, mainly for carbohydrates, such as NDF and starch, on performance of beef and dairy cattle (Oba and Allen, 1999; Vander Pol et al., 2008; Owens et al., 2016). In dairy cattle, Oba and Allen (1999) demonstrated that for each 1-unit improvement on NDF digestibility, dry matter intake, milk yield, and 4% fat-corrected milk yield increased by 0.17, 0.23, and 0.25 kg, respectively. Moreover, rumen starch digestibility has been positively related with total tract starch digestibility, dry matter intake, and milk yield (Ferraretto et al., 2013). In beef cattle, increasing starch degradability in the rumen by corn grain processing reduced fecal starch (Owens et al., 2016), while also improving average daily gain and feed efficiency (Vander Pol et al., 2008). Hence, nutritional technologies or strategies that maximize rumen nutrient digestibility, while also maintaining an adequate rumen function and health, are warranted to benefit ruminant productivity and overall profitability of the operation.

One of these promising nutritional technologies alternatives in the ruminant segment are the DFM, also known as probiotics, which are live micro-organisms that confer a health benefit to the host when offered in adequate amounts (FAO/WHO, 2001; Markowiak and Śliżewska, 2018). *Bacillus* spp. are one type of DFM, being classified as gram-positive, catalase-positive, spore-forming, aerobic, and facultative anaerobic bacteria (Luise et al., 2022). Besides its proven modes of action and benefits on health variables, such as pathogen inhibition (Copani et al., 2020), biofilm formation (Segura et al., 2020), and mucin formation (Santano et al., 2020), *Bacillus* spp. have also been known for its ability to produce a diverse range and quantities of enzymes of interest in ruminant nutrition, such as fibrolytic, amylolytic, proteolytic, and lipolytic (Schallmey et al., 2004; Elshaghabee et al., 2017; Luise et al., 2022). Recently, Pan et al. (2022) evaluated the effects of the same combination of strains used herein on in vitro DM and NDF digestibility of 10 forage sources originated from Australia. These authors reported a mean improvement of 13.1% and 27.6% on in vitro DM and NDF digestibility (6.0 and 8.1 percentage units, respectively) following DFM inoculation. Data from experiment 1 corroborate our previous research findings, as in vitro NDF digestibility increased by 12.6% at 24 h, whereas 48 h DM and NDF digestibility increased by 3.7 and 15.1%, respectively. Both, present and previous findings (Pan et al., 2022), further support that the combination of the *B. licheniformis* and *B. subtilis* has the potential to improve in vitro DM and NDF digestibility of different feedstuffs that contain >32% NDF (DM basis).

One additional point of interest of the current experiment was to evaluate how the DFM mixture containing *B. licheniformis* and *B. subtilis* would behave when ­commercial dairy TMR were used (experiment 2), as to the best of our knowledge, we are lacking studies evaluating the effects of DFM treatment on TMR. In agreement with the results from experiment 1, inoculation of a *Bacillus*-based DFM improved in vitro mean DM and NDF digestibility at 24 and 48 h by a range of 1.2 (+1.8%) to 4.0 (12.0%) percentage points. However, contrary to results observed earlier (Pan et al., 2022), no DFM effects were observed on in vitro starch digestibility of the different commercial dairy TMR. One may speculate that the amount of starch in the commercial dairy TMR was not as high (from 19.1% to 27.0%; DM basis) as observed in the individual feedstuffs analyzed by Pan et al. (2022), and it may have contributed to the lack of treatment differences observed herein. Additionally, previous in vitro unpublished data (personal communication) demonstrated that *Bacillus* spp. may alter its enzymatic expression based on substrate availability, through a mechanism known as induction effect, which could help to explain the different results between individual high-concentrate feedstuffs (Pan et al., 2022) when compared with the complete dairy TMR used in experiment 2. In fact, this induction mechanism has been demonstrated for another features and modes of action in *Bacillus* spp., such as biofilm formation against plant polysaccharides (Beauregard et al., 2013), as well as plant sucrose-stimulated root colonization, solid surface motility, and surfactin production (Tian et al., 2021).

Gas production can be used as an indirect measurement of carbohydrates degradability (Menke and Steingass, 1988; Getachew et al., 2005). In vitro gas production originates from microbial degradation of feed and buffering of acids produced during ruminal fermentation (Getachew et al., 2005). Gas production also reflects production of VFA, as when feed is fermented to acetate and butyrate, fermentative gas is produced while propionate synthesis yields gas by buffering of the acids (Blummel and Orskov, 1993; Getachew et al., 2005). In agreement with the observed effects on nutrient digestibility, DFM inoculation increased in vitro gas production in experiments 1 (24 and 48 h) and 2 (48 h only). Interestingly, data from experiment 1 were further included into the Menke and Steingass (1988) equation for estimation of metabolizable energy (**ME**; Mcal/kg) of the feedstuffs following CON and DFM treatment administration. Overall, our results demonstrate that mean ME of the feedstuffs was increased by 4.8% and 5.1% after 24 and 48 h of DFM inoculation, respectively. Metabolizable energy values were further used to estimate net energy for maintenance (**NE**_**m**_) and gain (**NE**_**g**_) (NRC, 1996; NASEM, 2016) and we observed greater improvements in NE_m_ and NE_g_ (averaged 6.0% and 8.1%, respectively) with DFM, which, subsequently has the potential to increase animal performance. Based on equations proposed and validated by others (Valadares Filho et al., 2016), improvements in feedstuff NE_g_ with DFM inoculation may result in an additional 32 g of carcass average daily gain (**cADG**) in a 450-kg body weight feedlot beef animal, leading to an approximate improvement of 60 g in ADG (considering a dressing percent = 53.0%). Supporting this calculation, Dias and colleagues (2022) recently observed a numerical improvement of 70 g/d in *B. indicus* feedlot bulls offered the same *B. licheniformis* and *B. subtilis* combination as the one evaluated herein.

Lastly, the combined analysis with single feedstuffs from Pan et al. (2022) was performed and demonstrated that the improvements on in vitro DM and NDF digestibility did not depend on forage quality based on median CP and NDF content of the feedstuffs, highlighting the fiber degrading ability of *Bacillus* spp., regardless of the type and quality of the feedstuff. *Bacillus* spp. secretes numerous enzymes, such as fibrolytic, amylolytic, and lipolytic (Schallmey et al., 2004), that may degrade a variety of substrates, enabling the bacterium to survive in a continuously changing environment (Su et al., 2020). This genus and some of its close relatives have excellent enzymatic secretion ability, making them important hosts to produce medicinal proteins, industrial enzymes, and according to Schallmey et al. (2004), enzymes produced using *B. subtilis* may account for roughly 50% of the total enzyme market. Also, significant moderate to strong correlations were observed between in vitro DM and NDF digestibility in both treatments and in both timepoints of the present study.

## CONCLUSIONS

In summary, inoculation of a *Bacillus*-based direct-fed microbial improved mean in vitro rumen fermentation traits (gas production, dry matter, and neutral detergent fiber digestibility) of single feedstuffs and commercial dairy TMR, but no differences were observed on in vitro starch digestibility. Based on findings from these in vitro studies, we can suggest that the *Bacillus*-based direct-fed microbial used herein (BOVACILLUS) may have potential to improve nutrient digestibility and subsequently performance of beef and dairy cattle. Nonetheless, additional studies are warranted to understand how different nutrients from the ruminant diets interact and impact the expression of the enzymes produced by *Bacillus* spp.
